# An Integrative Metabolomic and Network Pharmacology Study Revealing the Regulating Properties of Xihuang Pill That Improves Anlotinib Effects in Lung Cancer

**DOI:** 10.3389/fonc.2021.697247

**Published:** 2021-08-09

**Authors:** Chunyu Li, Zhihong Wang, Wei Chen, Bo Cao, Mingyu Zhang, Qiong Gu, Shuya Qi, Xiaofei Fei, Yafei Shi, Xingjie Li, RuiSheng Li, Jiabo Wang, Guohui Li

**Affiliations:** ^1^National Cancer Center/National Clinical Research Center for Cancer/Cancer Hospital, Chinese Academy of Medical Sciences and Peking Union Medical College, Beijing, China; ^2^Research Center for Clinical and Translational Medicine, Fifth Medical Center of Chinese People’s Liberation Army (PLA) General Hospital, Beijing, China; ^3^School of Traditional Chinese Medicine, Capital Medical University, Beijing, China

**Keywords:** Xihuang pill, anlotinib, lung cancer, untargeted metabolomics, network pharmacology, integrated strategy

## Abstract

Lung cancer ranks as a leading cause of death. Although targeted therapies usually trigger profound initial patient responses, these effects are transient due to drug resistance and severe side effects. Xihuang Pill (XHW) is a popular Chinese medicine formula that might benefit cancer patients when used as a complementary therapy. However, its underlying mechanism when combined with anticancer drugs is not clearly understood. Here, we used an integrated strategy to reveal the regulatory properties of XHW in increasing the antitumor activity of anlotinib in lung cancer. We evaluated the anti-lung cancer effect of XHW combined with anlotinib in mice bearing Lewis lung carcinoma (LLC). We applied untargeted metabolomics to identify the differences metabolism and found that XHW improved the effects of anlotinib on lung cancer. The components and targets related to the effects of XHW treatment on lung cancer were obtained through network pharmacology. Then, by integrating the biologically active components of XHW and anlotinib as well as the treatment-responsive metabolites and their related targets, an interaction network was constructed to evaluate the combination therapy. Finally, important protein candidates for this response were verified by immunohistochemistry of tumor tissues. The results showed that XHW significantly improved the inhibitory effect of anlotinib on tumor growth in LLC-bearing mice. Additionally, 12 differentially-abundant metabolites were identified by untargeted metabolomics in the XHW/anlotinib group compared with the XHW or anlotinib groups, and they were mainly enriched in fatty acid metabolism, lipid metabolism and amino acid metabolism pathways. Anlotinib, 23 components in Shexiang, 2 components in Niuhuang, 30 components in Ruxiang and 60 components in Moyao work together to act on 30 targets to regulate hexadecanoic acid (also named palmitic acid), linoleic acid, lactosylceramide, adrenaline, arachidonic acid and lysoPC(18:1(9Z)). The results of immunohistochemistry showed that XHW combined with anlotinib reduced the expression of PDGFRA in tumors. Overall, the key metabolites of XHW that enhances the efficacy of anlotinib were regulated by a multicomponent and multitarget interaction network. Our results suggested that anlotinib combined with XHW may be a promising strategy for the treatment of lung cancer.

## Introduction

Lung cancer remains the leading cause of cancer-related death worldwide, and its 5-year survival rate is less than 20% (1). Non-small cell lung cancer (NSCLC) accounts for approximately 80-85% of all diagnosed cases. The discovery of epidermal growth factor receptor tyrosine-kinase inhibitors (EGFR-TKIs) was a breakthrough that established a paradigm of precision medicine for the treatment of EGFR-mutant NSCLC over the past few decades. Anlotinib is a novel multitargeted TKI that has been approved by the China Food and Drug Administration (CFDA) and State Food and Drug Administration (SFDA) (2), and phase III clinical trials suggest that anlotinib can significantly prolong overall survival (OS) and progression free survival (PFS) of NSCLC patients (3). However, acquired resistance to EGFR-TKIs inevitably develops in tumors at later stages, regardless of whether the third-generation TKIs are osimertinib or anlotinib (4, 5). Therefore, new strategies are needed for the treatment of NSCLC to achieve longer and deeper remissions.

Traditional Chinese medicine (TCM) has been widely applied as an alternative and complementary therapy for cancer patients in China (6). Xihuang Pill (XHW) was first recorded in the ‘surgical syndrome all-life set’ for the treatment of breast cancer, lung cancer and colorectal cancer (7), and it is composed of Bovis calculus (*Bos taurus domesticus* Gmelin, Niuhuang in Chinese), Moschus (*Moschus berezovskii* Flerov, *Moschus sifanicus* Przewalski, *Moschus moschiferus* Linnaeus, Shexiang in Chinese), Olibanum (*Boszvellia carterii* Birdw., *Boswcllia bhaurdajiana* Birdw., Ruxiang in Chinese) and Myrrha (*Commiphora myrrha* Engl., *Commiphora molmol* Engl., Moyao in Chinese). Reports have suggested that XHW inhibits tumor growth, reduces the toxicity and side effects of chemotherapy, enhances the efficacy of chemotherapy, prolongs survival time and improves the quality of life of patients with breast cancer, lung cancer, gastrointestinal tumors, or colon tumors (8–10). Thus, the combination of XHW with anlotinib may be a promising therapeutic strategy to improve the efficacy of anlotinib. Moreover, a greater understanding of the modulatory mechanism of XHW to enhance the effect of anlotinib could help to address this unmet medical need.

In this study, metabolomics and network pharmacology were used to gain insights into the modulatory role of XHW to enhance the efficacy of anlotinib in the treatment of lung cancer. We chose an LLC-bearing mouse model to substantiate our integration strategy by examining whether the combination of XHW anlotinib indeed inhibited tumor growth. Furthermore, we established a comprehensive network of compound-target-metabolite associations to address unanswered questions related to the regulatory role of XHW in enhancing the effects of anlotinib. This strategy can provide a theoretical basis for research on XHW treatment combined with anlotinib for lung cancer therapy.

## Materials And Methods

### Cell Lines, Reagents, and Drugs

Lewis lung cancer (LLC) cells were supplied by Cell Bank of the Chinese Academy of Sciences (Shanghai, China). Dulbecco’s modified Eagle’s medium (DMEM), antibiotic-antimycotic solution, and fetal bovine serum (FBS) were purchased from GIBCO BRL-Life Technologies, Inc (Grand Island, NY, USA). Anti-PDGFRA antibody (ab124392) was obtained from Abcam (Cambridge, MA, USA). Goat anti-rabbit IgG was purchased from Jackson ImmunoResearch (West Grove, PA, USA). HPLC-grade acetonitrile and methanol were acquired from Merck (Darmstadt, Germany) and Fisher Chemicals (Pittsburg, PA, USA), respectively. Purified water was prepared with a Millipore ultrapure water system (Millipore, Bedford, MA, USA). Other reagents and chemicals were commercially available. Xihuang Pill (Lot number: 1910002) was produced by Zhejiang Tianyitang Pharmaceutical Co., Ltd, China. Anlotinib hydrochloride was purchased from CHIA TAI TIANQING PHARMACEUTICAL GROUP Co., LTD, China. Anlotinib was dissolved in saline and stored in the dark at -20°C.

### Cell Culture and Animal Handling

LLC cells were cultured in DMEM supplemented with 1% (v/v) antibiotic-antimycotic and 10% (v/v) fetal bovine serum at 37°C under a humidified atmosphere containing 5% CO_2_. Male C57BL/6N mice (weighing 20 ± 5 g) were supplied by SPF (Beijing) Biotechnology Co., Ltd. (License No. SCXK 2019-0010). All animals had free access to drinking water and food. Standard breeding conditions were maintained during the whole experiment, with a temperature of 25 ± 2°C, relative humidity of 50-60%, and 12 h/12 h light/dark cycle. The whole experiment was performed in accordance with the NIH Guide for the Care and Use of Laboratory Animals approved by the Scientific Investigation Board of Cancer Hospital, Chinese Academy of Medical Sciences and Peking Union Medical College.

### Tumor Model and Sample Collection

We previously reported that XHW significantly inhibited tumor growth in mice bearing Lewis lung carcinoma (LLC) in a dose-dependent manner (11). Here, we aimed to investigate the combined efficacy of XHW and anlotinib on tumor growth. Therefore, only one dosage of anlotinib or XHW and one-time point were used in this study. The optimal doses of XHW (0.78 g/kg) and anlotinib (1 mg/kg) were determined in the combined treatment according to our preliminary experimental results. The duration of the experiment was 21 days, because the treatment of anlotinib was 21 days per cycle. Mice were randomly separated into the following groups (N=9-10 in each): normal group, model group, anlotinib-treated group, XHW-treated group and combination XHW with anlotinib-treated group. For all except the normal group, 0.1 ml LLC cells (1 × 10^7^/mL) were injected subcutaneously into the right dorsal region of the mice to induce lung cancer. One day after LLC inoculation, the mice were orally administered 1 mg/kg anlotinib and 0.78 g/kg XHW for 21 consecutive days, either alone or in combination. The normal group and the model group were given an equivalent volume of normal saline. Body weight of the mice was measured every 3 days. Mice were fasted for 24 h after drug administration on day 21. Blood was collected retro-orbitally and centrifuged at 3500 rpm for 10 min. Mice were sacrificed by intraperitoneal injection of 5% pentobarbital sodium. Then, the heart, liver, spleen, lung, kidney and tumor were removed and weighed. The tumor volume was measured and calculated by the following formula: length × width ^2^/2.

### Histological Examination

The heart, liver, spleen, lung, and kidney were fixed in 10% neutral buffered formalin. Then, tissues were paraffin embedded, cut into 4 µm sections and stained with hematoxylin and eosin (H&E) as described previously (12). Histopathological sections were evaluated using four distinct semiquantitative grades according to the grading systems of the International Harmonization of Nomenclature and Diagnostic Criteria (INHAND) for Lesions in Rats and Mice (**Supplementary Table S1**) (13).

### Immunohistochemistry Analysis

The immunohistochemistry analysis was performed by following the previously reported protocols (14). Briefly, OCT-embedded frozen samples were cut into 10-μm thick sections. Cryosections were incubated overnight at 4°C with primary mouse antibody against PDGFRA (1:100). The following day, sections were washed with PBS and then incubated for 30 minutes at 37°C with secondary antibodies. After washing, slides were incubated with 3,3-diaminobenzidine tertrahydrochloride (DAB) for color development. The immunostained tumors (positive areas) were examined using light microscopy with a digital camera system and analyzed using Image-Pro Plus 6.0.

### Sample Preparation for Metabolomics

Sample preparation for metabolomics was performed according to our previous method (15). The plasma samples were thawed, and aliquots were mixed with cold methanol at a ratio of 1:3 (v:v). The mixtures were vortexed for more than 30 s, and then centrifuged at 14,000 rpm for 10 min at 4°C. Then, 120 µL of the supernatant was transferred to a clean centrifuge tube, spin-dried and then re-dissolved in 60 µL of 75% of methanol, vortexed, and centrifuged once again. Finally, each supernatant was stored at -80°C prior to further analysis. A quality control (QC) sample was prepared with an aliquot of 4 μL of each sample and was used to validate the stability and repeatability of the system.

### Conditions of Chromatography and Mass Spectrometry

Liquid chromatographic analysis was carried out using LC-IT-TOF/MS (Shimadzu Corp., Japan). A Inertsil ODS-SP C18 (4.6×150 mm, 5 μm) column was used and the column temperature was held at 40°C. The detection wavelength was 275 nm. The sample injection sequence was random and the injection volume was 15 μL. The autosampler temperature was maintained at 4°C throughout the analysis. The mobile phase included solvent A (water) and solvent B (acetonitrile). The elution programme was as follows: 0-0.01 min, 5% B; 0.01-3 min, 5%-12% B; 3-7 min, 12%- 30% B; 7-10 min, 30% - 50% B; 10-14 min, 50% - 75% B; 14-25 min, 75% -95% B; 25-31 min, 95% -95% B; and 31-31.01 min, 95%-5% B. The flow rate was set at 1 mL/min.

Mass spectrometry was performed on an LC-IT-TOF/MS system with an electrospray ionization source (ESI) running in both positive (ESI+) and negative (ESI-) modes. MS data was gathered in full scan mode with a range of m/z 100-1000. The dry gas temperature was set to 200°C (ESI-) and 225°C (ESI+), and its flow rate was 1.5 L/min. The nozzle voltage was 500 V in both ESI- and ESI+. The electrospray capillary voltage was 3.5 kV (ESI-) and 4.5 kV (ESI+).

### Data Extraction and Analysis

The raw data were uploaded to MetaboAnalyst 3.0 (http://www.metaboanalyst.ca/) and normalized. Next, the resultant data were subjected to multivariate statistical analysis using SIMCA-P 14.1 software (Umetrics, Umea, Sweden). Unsupervised principal component analysis (PCA) was carried out to visualize data with sample classes and assess metabolome changes. To screen differential metabolites between the two groups, orthogonal partial least squares-discriminant analysis (OPLS-DA) was used as a supervised multivariate model. Variable importance in projection (VIP) values were calculated to determine the key mass ions, which contributed to distinguishing two orthogonal comparisons in the OPLS-DA model. Meanwhile, S-plots generated to avoid false positive results, and variables plotted at the top or bottom were considered the most important metabolites. Thus, only mass ions with VIP values > 1 and |p(corr)| ≥ 0.5 were ultimately considered be discriminant critical characteristic metabolites. Moreover, a t-test was performed in parallel using MetaboAnalyst 3.0 and mass ions with *P* < 0.05 and fold change > 2 or < 0. 5 were selected for further study. Then, area-proportional 3-Venn diagrams were used to search for the common metabolites affected by the synergistic effect of the two drugs. Subsequently, the molecular formulas of the above metabolites were identified by using METLIN (http://metlin.scripps.edu) and HMDB (http://www.hmdb.ca/). Finally, the acquired metabolites were uploaded to MetaboAnalyst 3.0 for pathway enrichment analysis and visualization of the affected pathways. Metabolites enriched in these pathways were considered to be the most important biomarkers.

### Network Pharmacology Analysis

Potential biomarkers associated with the ability of XHW to enhance the effects of anlotinib against lung cancer were identified and then imported into the MBRole 2.0 database (http://csbg.cnb.csic.es/mbrole2/) to obtain their protein targets. Additionally, their UniProt IDs were acquired by uniformly converting protein ID types. The targets of anlotinib were from the literature (2, 16, 17), while the targets of XHW were obtained according to our previously described method (11). Protein-protein interaction (PPI) analyses were performed to connect this network with the biomarker-protein network to construct a “compound-target-metabolite” network. Ultimately, the PPI network was visualized using Cytoscape 2.8.3 (National Institute of General Medical Sciences, United States).

### Statistical Analysis

Raw data are presented as mean ± SE. GraphPad Prism 8.0 was used to perform one-way analysis of variance (ANOVA). Statistically significant differences were set at P < 0.05, and highly significant differences were set at P < 0.01.

## Results

### Efficacy of Anlotinib and XHW in the LLC Model

**Figure 1:**
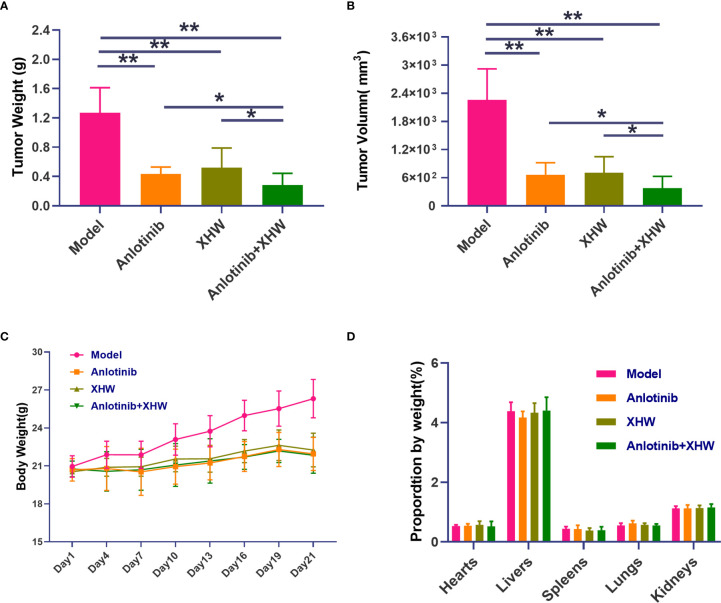
Effect of cotreatment with anlotinib and XHWon the LLC mouse model. **(A)** Tumor weight, **(B)** tumor volume, **(C)** organ weight, and **(D)** body weight changes during the 21 days of the experiment. Data are mean ± SD. P values are based on ANOVA, **P<0.01 versus model, *P<0.05 versus model. N = 9–10 in each group.

**Figure 2:**
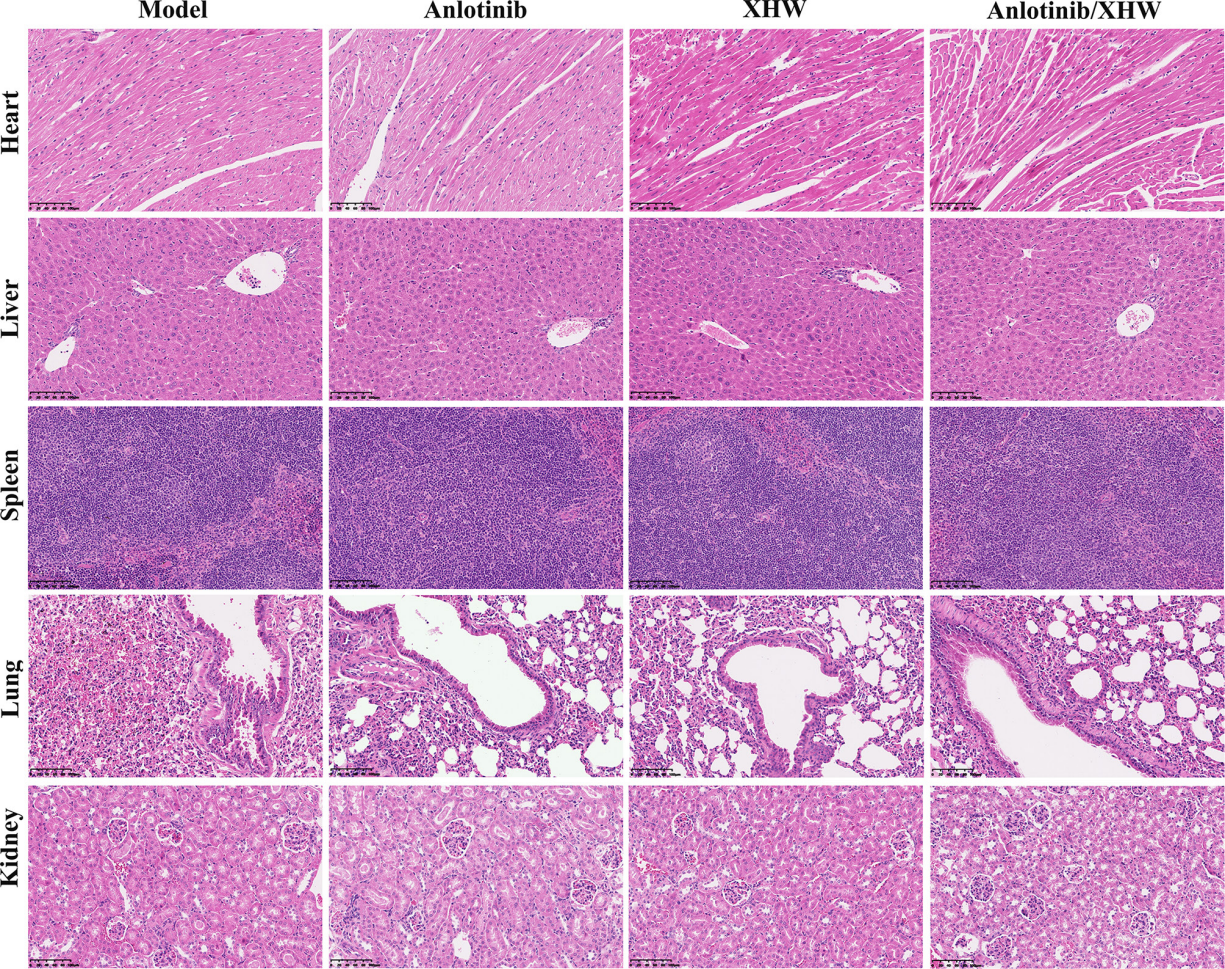
Representative microphotographs of the heart, liver, spleen, lung, and kidney of the mice bearing LLC after treatment for 21 days.

To explore the antitumor activity of anlotinib combined with XHW *in vivo*, an LLC model was established. As shown in **Figures 1A, B**, compared with the normal group, treatment with anlotinib or XHW alone significantly suppressed tumor growth based on tumor mass and volume measured on day 21 after treatment (P<0.01). The combination of anlotinib with XHW showed stronger tumor inhibition than anlotinib or XHW alone (P <0.05), with effects 1.18 and 1.32 times stronger than those of anlotinib and XHW alone, respectively. Body weight was measured every 3 days throughout the experiment, and the results demonstrated no significant weight loss in any group (**Figure 1C**). Moreover, no obvious changes were observed about the proportion by weight of heart, liver, spleen, lung and kidney between different groups (**Figure 1D**). As shown in **Figure 2**, the histopathological results of the heart, liver, spleen, lung, and kidney showed that, compared with the model group, there was no aggravation of the lesions in the combined treatment group or in either single-drug treatment group. **Supplementary Table S2** shows the histopathological scoring results of these tissues by three pathologists.

### Multivariate Statistical Analysis

**Figure 3:**
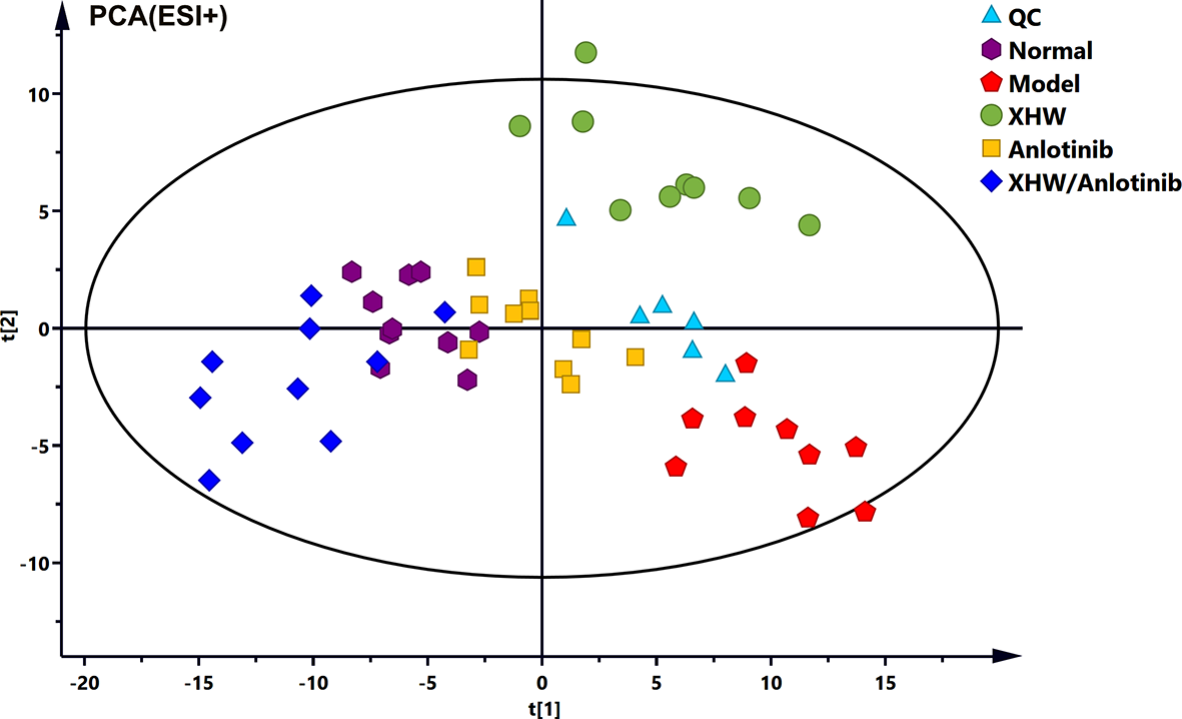
(PCA score plots of the combination of anlotinib with XHW against lung cancer in ESI+ mode.

**Figure 4:**
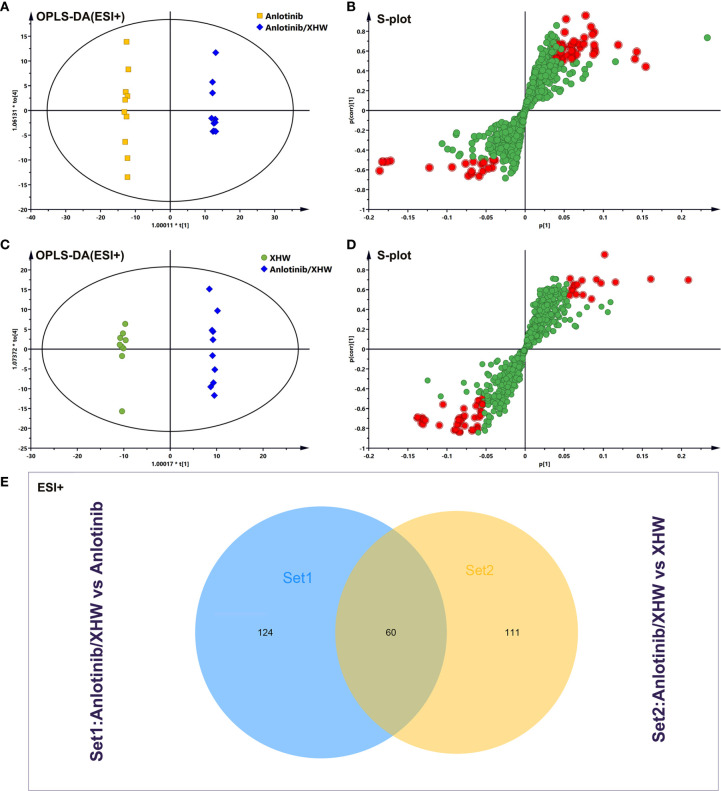
Analysis of potential biomarkers associated with anlotinib plus XHW in the treatment of lung cancer in ESI+ mode. **(A)** Results of the OPLS-DA model using the data from the anlotinib/XHW group *vs* the anlotinib group. **(B)** S-score plot constructed from the supervised OPLS analysis of the anlotinib/XHW group *vs* the anlotinib group. **(C) **Results of the OPLS-DA model using the data from the anlotinib/XHW group *vs* the XHW group. **(D)** S-score plot constructed from the supervised OPLS analysis of the anlotinib/XHW group *vs* the XHW group. Metabolite ions with variable influence on the projection (VIP) values >1 and |p(corr)| ≥ 0.5 are marked with a red square. The x-axis represents the co-correlation coefficient between the principal component and the metabolite, and the y-axis represents the correlation coefficient between the principal component and the metabolite. **(E) **Venn diagram of metabolites showing the overlap between the anlotinib/XHW, anlotinib, and XHW groups.

To investigate the changes in the metabolic profile of XHW that might potentiate the antitumor effect of anlotinib and to identify potential important metabolites, plasma metabolites extracted from each group were analyzed in both ESI+ and ESI- modes. First, unsupervised multivariate statistics (PCA) were performed, and the results are shown in **Figure 3** (ESI+) and **Supplementary Figure S1** (ESI-). A tendency to separate the anlotinib/XHW group, the XHW group, the model group, and the normal group was observed in both PCA score plots. An orthogonal partial least squares discrimination analysis (OPLS-DA) was then conducted to maximize the intergroup differences and aid in the identification of metabolites responsible for class separation. In the OPLS-DA score plot of the ESI+ mode, a clear separation between the anlotinib/XHW and anlotinib groups was observed (**Figure 4A**), with a Q2 of 0.518, an R2X of 0.531, and an R2Y of 0.999. The anlotinib/XHW and XHW groups were discriminated based on Q2, R2X and R2Y values of 0.702, 0.559, and 0.998, respectively (**Figure 4C**). The R2 and Q2 in the paired group were greater than 0.5; thus, the OPLS-DA model was acceptable. The OPLS-DA score plot for the ESI- mode is displayed in **Supplementary Figures S2A, C**. To highlight the metabolite differences between the anlotinib/XHW, anlotinib, and XHW groups, S-plots were constructed following OPLS-DA analysis and are shown in **Figures 4B, D** (ESI+) and **Supplementary Figures S2B, D** (ESI-), respectively.

### Identification of Candidate Metabolites of Anlotinib Combined With XHW in the Treatment of Lung Cancer

**Figure 5:**
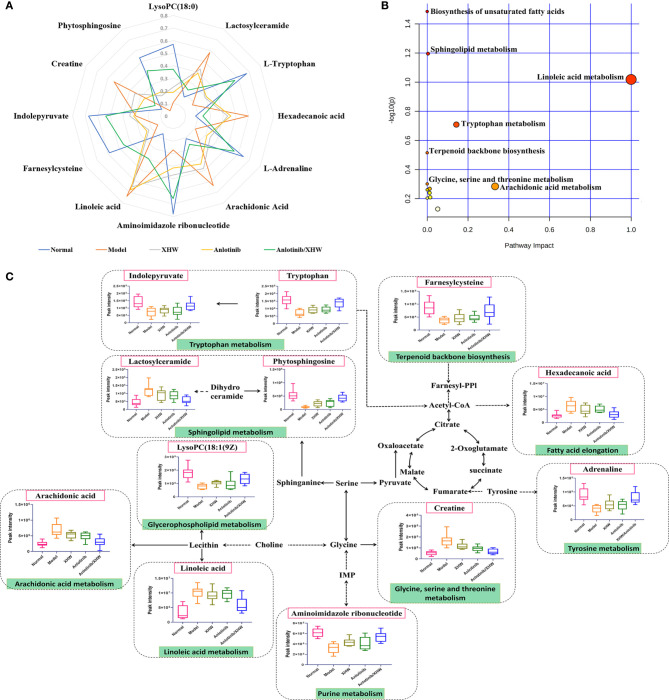
Overall metabolic profile. **(A)** Spider plot of 12 metabolites for which the change in relative abundance best differentiated the five experimental groups. **(B)** Schematic diagram of the affected metabolic pathways. **(C)** Network map of metabolic pathways and metabolites.

Based on the OPLS-DA analysis, the variables with VIP values > 1, |p(corr)| ≥ 0.5, P value < 0.05, and fold change > 2 or < 0. 5 were considered as candidate metabolites. To analyze similarities and differences between metabolites in two different groups, area-proportional 3-Venn diagrams was used. The results of **Figure 4E** show that 184 (anlotinib/XHW *vs* anlotinib) and 171 (anlotinib/XHW *vs* XHW) differentially abundant metabolites were detected in ESI+ mode, while 335 and 79 metabolites were detected for the same comparisons in ESI-mode, respectively (**Supplementary Figure S2E**). Thus, there were 60 common metabolites in ESI+ mode and 34 in ESI- mode that were affected by the synergistic effect of anlotinib and XHW, but not by anlotinib or XHW alone. Thus, these 94 metabolites could be used as potential biomarkers and were tentatively identified with the accurate mass charge ratio. As a result, 64 metabolites were ultimately identified (**Supplementary Table S3**). To further explore the altered metabolic pathways, these 64 metabolites were imported into MetaboAnalyst 3.0 for pathway enrichment analysis. Consequently, 12/64 differential metabolites were identified, including tryptophan, adrenaline, indolepyruvate, aminoimidazole ribonucleotide, farnesylcysteine, phytosphingosine, hexadecenoic acid (also named palmitic acid), creatine, lactosylceramide, lysoPC(18:1(9Z)), linoleic acid and arachidonic acid (**Table 1**). To more intuitively describe the changing trends between each group, a spider diagram was produced depicting the relative abundance of 12 metabolites. As shown in **Figure 5A**, except for lactosylceramide, hexadecenoic acid, creatine, linoleic acid, and arachidonic acid, the levels of the other metabolites in the model group were significantly lower than in the normal group. In addition, compared with the model group, the levels of lactosylceramide, hexadecenoic acid, creatine, linoleic acid, and arachidonic acid were decreased in the anlotinib/XHW group. In contrast, the levels of tryptophan, indolepyruvate, farnesylcysteine, lysoPC(18:1(9Z)), aminoimidazole ribonucleotide, adrenaline, and phytosphingosine were increased. Therefore, the levels of these metabolites in the combination group were consistent with those of the normal group.

**Table 1:** Differential metabolites and the corresponding pathways.

No.	Retention time(min)	Metabolites	m/z	Model *vs* Normal	Anlotinib/XHW *vs* Model	Pathways
1	3.245	Aminoimidazole ribonucleotide	295.06	↓	↑	Purine metabolism
2	3.36	Creatine	131.07	↑	↓	Glycine, serine and threonine metabolism Arginine and proline metabolism
3	3.5	Adrenaline	183.09	↓	↑	Tyrosine metabolism
4	3.736	Indolepyruvate	203.06	↓	↑	Tryptophan metabolism
5	7.986	Tryptophan	204.09	↓	↑	Tryptophan metabolism
						Aminoacyl-tRNA biosynthesis
6	22.693	Lactosylceramide	805.56	↑	↓	Sphingolipid metabolism
7	20.754	LysoPC 18:1(9Z)	521.35	↓	↑	Glycerophospholipid metabolism
8	22.148	Arachidonic acid	304.24	↑	↓	Biosynthesis of unsaturated fatty acids Arachidonic acid metabolism
9	22.678	Farnesylcysteine	325.21	↓	↑	Terpenoid backbone biosynthesis
10	22.709	Linoleic acid	280.24	↑	↓	Biosynthesis of unsaturated fatty acids linoleic acid metabolism
11	23.906	Phytosphingosine	317.29	↓	↑	Sphingolipid metabolism
12	24.225	Palmitic acid (Hexadecanoic acid)	256.24	↑	↓	Biosynthesis of unsaturated fatty acids
						Fatty acid elongation
						Fatty acid degradation
						Fatty acid biosynthesis

KEGG functional enrichment analysis demonstrated that the identified metabolites are mostly involved in biosynthesis of unsaturated fatty acids, sphingolipid metabolism, linoleic acid metabolism, tryptophan metabolism, terpenoid backbone biosynthesis, glycine, serine and threonine metabolism, glycerophospholipid metabolism, arachidonic acid metabolism, arginine and proline metabolism, fatty acid elongation, fatty acid degradation, tyrosine metabolism, fatty acid biosynthesis, aminoacyl-tRNA biosynthesis, and purine metabolism. These many terms were classified as fatty acid metabolism, lipid metabolism, and amino acid metabolism (**Table 1**). Hexadecenoic acid, linoleic acid, and arachidonic acid are all involved in biosynthesis of unsaturated fatty acids. Hexadecenoic acid annotations include fatty acid elongation, fatty acid degradation, and fatty acid biosynthesis. Linoleic acid annotations include linoleic acid metabolism. Arachidonic acid annotations include arachidonic acid metabolism. The top seven pathways were biosynthesis of unsaturated fatty acids, sphingolipid metabolism, linoleic acid metabolism, tryptophan metabolism, terpenoid backbone biosynthesis, glycine, serine and threonine metabolism, and arachidonic acid metabolism (**Figure 5B**). Moreover, to compare the differences in metabolic profiles among all experimental groups, a complex network was established according to KEGG pathways and the 12 significant metabolites (**Figure 5C**). The biosynthesis of unsaturated fatty acids pathway is not shown in **Figure 5C** because it is the general name of fatty acid metabolic pathways.

### “Compound-Target-Metabolite” Network Construction and Analysis

**Figure 6:**
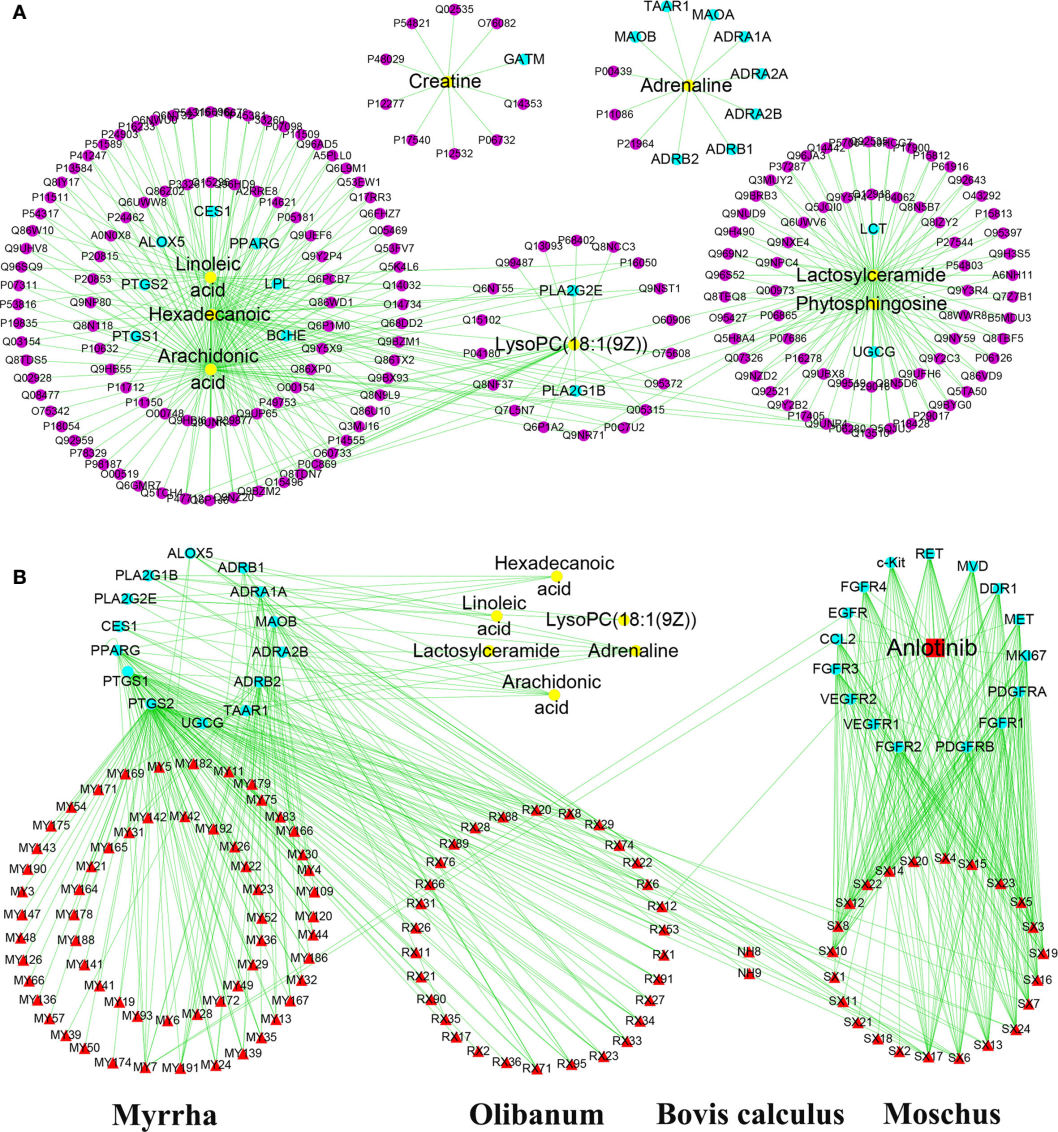
“Compound-target-metabolite” interactive network construction for the effects of anlotinib and XHW in the treatment of lung cancer. **(A)** Direct target information regulating metabolites. **(B)** Anlotinib and the chemical components of Xihuang Pills coregulate the metabolite network through direct targets. The purple dots represent the targets associated with potential metabolites. The yellow dots represent potential metabolites. The red square and red triangles represent anlotinib and the chemical components of XHW, respectively.

To explore the regulatory effects of XHW and anlotinib on the 12 metabolites, a “compound-target-metabolite” interaction network was constructed. We found that XHW improved the efficacy of anlotinib through multiple components and targets. XHW played a major role in regulating metabolism. Furthermore, the main ingredients from Shexiang in Xihuang Pill act directly on the targets of anlotinib. Specifically, 8 potential metabolites, such as lysoPC(18:1(9Z)) (C04230), linoleic acid (C01595), hexadecanoic acid (C00249), arachidonic acid (C00219), phytosphingosine (C12144), adrenaline (C00788), lactosylceramide (C01290), and creatine (C00300), participated in the interaction network. As shown in **Figure 6A** and **Table 2**, the following 20 drug targets regulated the 8 potential metabolites directly: BCHE, ALOX5, PTGS2, PTGS1, LPL, CES1, PLA2G2E, PLA2G1B, UGCG, PPARG, LCT, ADRA1A, ADRB1, ADRB2, MAOB, ADRA2A, MAOA, TAAR1, ADRA2B, and GATM. The results of **Figure 6B** and **Table 2** show that anlotinib, 23 components in Shexiang, RX12((R)-linalool) in Ruxiang and MY7(quercetin) in Moyao work together on 16 targets: c-Kit, FGFR4, EGFR, CCL2, FGFR3, VEGFR2, VEGFR1, FGFR2, PDGFRB, FGFR1, PDGFRA, MKI67, MET, DDR1, MVD, and RET. Two components in Niuhuang, 8 components in Shexiang, 30 components in Ruxiang and 60 components in Moyao directly regulate hexadecanoic acid, linoleic acid, lactosylceramide, adrenaline, arachidonic acid and lysoPC(18:1(9Z)). Six components in Shexiang, 29 components in Ruxiang, 54 components in Moyao and 2 components in Niuhuang act on the following targets involved in the regulation of arachidonic acid: ALOX5, PLA2G1B, PLA2G2E, CES1, PPARG, PTGS1 and PTGS2. Further, 9 components in Ruxiang and 10 components in Moyao act on PPARG; SX13, and RX22 act on PLA2G1B, SX7, SX13 and NH8 act on CES1, and SX6 acts on PLA2G2E to regulate hexadecanoic acid. SX13 and RX22 act on PLA2G1B and SX6 acts on PLA2G2E to regulate lysoPC(18:1(9Z)) together. SX13 and RX22 act on PLA2G1B, SX6 acts on PLA2G2E, and NH9, MY7, MY19, SX1, SX6, and SX11 act on ALOX5 to regulate linoleic acid. Moreover, 25 constituents in Moyao, 12 constituents in Ruxiang, and SX16 participate in regulating adrenaline by acting on ADRB1, ADRA1A, MAOB, ADRA2B, ADRB2, and TAAR1. SX17 acts on UGCG to regulate lactosylceramide.

**Table 2:** Key direct targets of anlotinib combined with XHW in the treatment of lung cancer.

No.	Gene names	Protein names	UniProt ID
1	BCHE	Cholinesterase	P06276
2	ALOX5	Arachidonate 5-lipoxygenase	P09917
3	PTGS2	Prostaglandin G/H synthase 2	P35354
4	PTGS1	Prostaglandin G/H synthase 1	P23219
5	LPL	Lipoprotein lipase	P06858
6	CES1	Liver carboxylesterase 1	P23141
7	PPARG	Peroxisome proliferator-activated receptor gamma	P37231
8	PLA2G2E	Group IIE secretory phospholipase A2	Q9NZK7
9	PLA2G1B	Phospholipase A2	P04054
10	UGCG	Ceramide glucosyltransferase	Q16739
11	LCT	Lactase-phlorizin hydrolase	P09848
12	ADRA1A	Alpha-1A adrenergic receptor	P35348
13	ADRB1	Beta-1 adrenergic receptor	P08588
14	ADRB2	Beta-2 adrenergic receptor	P07550
15	MAOB	Amine oxidase [flavin-containing] B	P27338
16	ADRA2A	Alpha-2A adrenergic receptor	P08913
17	MAOA	Amine oxidase [flavin-containing] A	P21397
18	TAAR1	Trace amine-associated receptor 1	Q96RJ0
19	ADRA2B	Alpha-2B adrenergic receptor	P18089
20	GATM	Glycine amidinotransferase, mitochondrial	P50440
21	c-Kit	Mast/stem cell growth factor receptor Kit	P10721
22	FGFR4	Fibroblast growth factor receptor 4	P22455
23	EGFR	Epidermal growth factor receptor	P00533
24	CCL2	C-C motif chemokine 2	P13500
25	FGFR3	Fibroblast growth factor receptor 3	P22607
26	VEGFR2,	Vascular endothelial growth factor receptor 2	P35968
27	VEGFR1,	Vascular endothelial growth factor receptor 1	P17948
28	FGFR2	Fibroblast growth factor receptor 2	P21802
29	PDGFRB,	Platelet-derived growth factor receptor beta	P09619
30	FGFR1	Fibroblast growth factor receptor 1	P11362
31	PDGFRA	Platelet-derived growth factor receptor alpha	P16234
32	MKI67	Proliferation marker protein Ki-67	P46013
33	MET	Hepatocyte growth factor receptor	P08581
34	DDR1	Regulatory protein E2	P08345
35	MVD	Diphosphomevalonate decarboxylase	P53602
36	RET	Proto-oncogene tyrosine-protein kinase receptor Ret	P07949

### Experimental Validation

**Figure 7:**
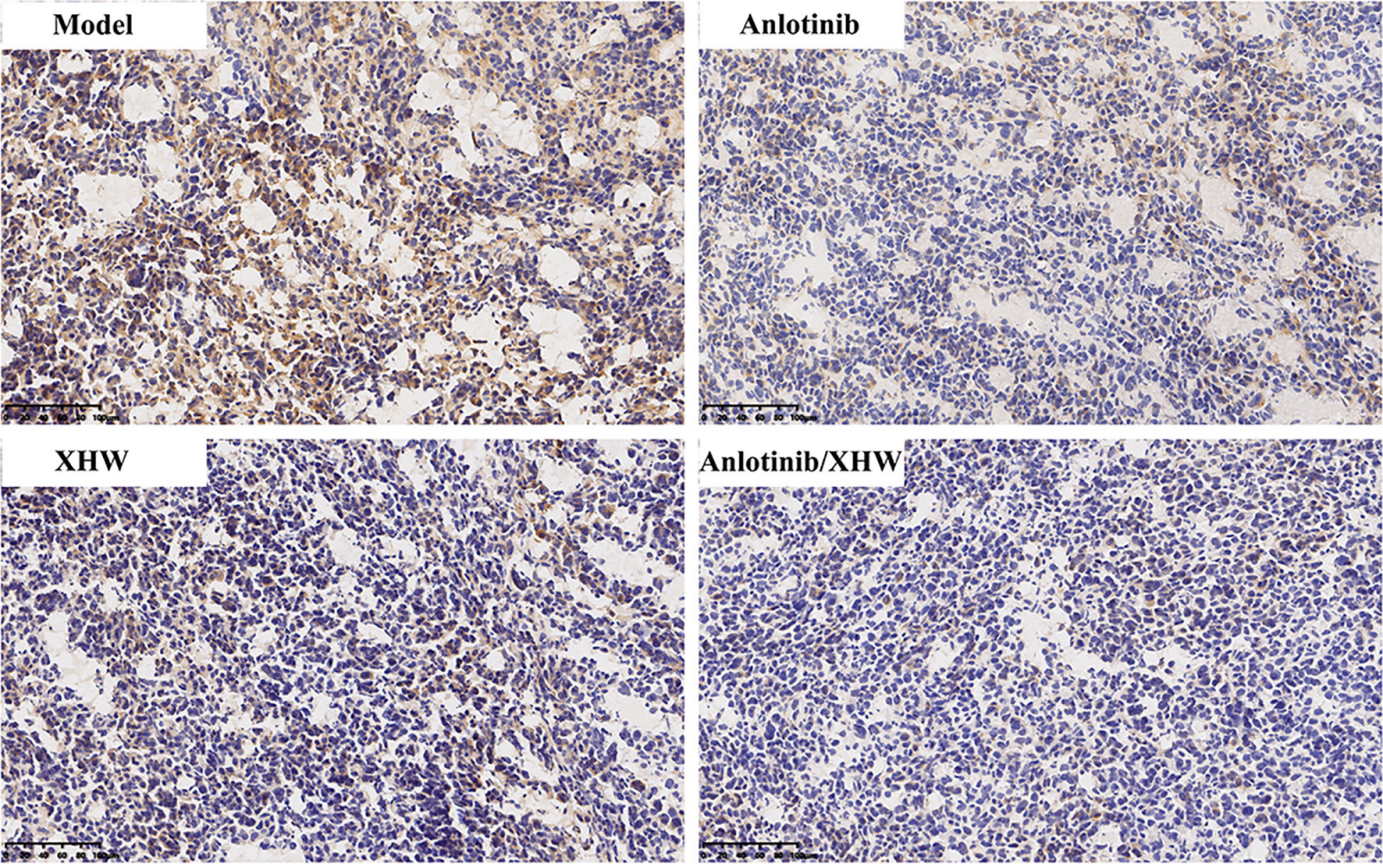
Effects of anlotinib and XHW on the expression of PDGFRA in the treatment of lung cancer by immunohistochemistry analysis.

To verify the authenticity of the combined metabolomics and network pharmacology results, immunohistochemistry analysis was used to determine the effect of anlotinib combined with XHW on PDGFRA in lung tumors. As shown in **Figure 7**, compared with the model group, PDGFRA levels in the anlotinib or XHW group exhibited a downward trend, and those in the anlotinib/XHW group were significantly decreased.

## Discussion

As a novel multitarget receptor tyrosine kinase inhibitor, anlotinib significantly prolongs the overall survival (OS) and progression-free survival (PFS) of NSCLC patients. However, anlotinib resistance inevitably occurs at later stages. The experimental results described here show that XHW can effectively enhance anlotinib to inhibit lung cancer growth and without any toxic side effects, indicating that anlotinib combined with XHW may be used to develop novel therapeutic strategies aimed at improving the prognosis of patients resistant to anlotinib.

A previous report showed that fatty acids are significantly related to a higher risk of NSCLC and could be regarded as predictive biomarkers (18, 19). Moreover, fatty acids play a key role in cancer cell proliferation (20). In this study, metabolomics was used to investigate metabolomic changes associated with phenotypic characterization of lung cancer treated with XHW plus anlotinib. Our data demonstrated that hexadecenoic acid, linoleic acid, and arachidonic acid were enriched in five fatty acid pathways, the most important of which is biosynthesis of unsaturated fatty acids. Compared with the model group, XHW plus anlotinib significantly reduced plasma levels of fatty acids such as linoleic acid, arachidonic acid, and hexadecenoic acid to levels similar to those in the normal group. A pilot study indicated that plasma levels of linoleic acid were significantly higher in lung cancer patients with EGFR mutations and identified linoleic acid as a potential biomarker for the early detection of lung cancer (21). Lung tumor cell survival can be inhibited by blocking arachidonic acid metabolism; thus, targeting arachidonic acid action may be beneficial for cancer therapy (22). High plasma levels of palmitic acid were associated with life expectancy for lung cancer patients, which could be used for early lung cancer diagnosis (23–25). Our results suggest that XHW improves the efficacy of anlotinib by restoring endogenous fatty acid homeostasis. Therefore, a combination of hexadecanoic acid, linoleic acid, and arachidonic acid may be a promising biomarker panel for monitoring the efficacy of XHW combined with anlotinib in the treatment of lung cancer.

Apart from regulating fatty acid metabolism, XHW plus anlotinib also affected lung cancer by modulating lipid metabolism and amino acid metabolism. Lipid metabolism is involved in various malignant processes and is currently considered to be a hallmark of cancer (26). Fatty acids are the main products in the process of *de novo* lipid synthesis and act as building blocks for the rapid growth of cancer cells (27). Sphingolipids play a key role in regulating cancer cell signals to control tumor suppression or survival and targeting sphingolipid metabolism may be a potential strategy for clinical anticancer treatment (28). In addition, tryptophan metabolism is a common therapeutic target for cancer and used in the development of clinical drugs (29, 30). Based on our results, we speculate that fatty acid metabolism, sphingolipid metabolism, and tryptophan metabolism may be therapeutic targets of XHW combined with anlotinib in the treatment of lung cancer.

The relationships among compounds, targets, and potential metabolites showed that XHW or anlotinib regulated the candidate metabolites directly or indirectly through a complex interaction network. Anlotinib is a small-molecule multitarget tyrosine kinase inhibitor that can effectively inhibit VEGFR, PDGFR, FGFR, c-Kit, and other kinases and that has antitumor angiogenic effects and can inhibit tumor growth. Interestingly, the main ingredients from Shexiang in XHW act on the targets of anlotinib. This result indicated that Shexiang in XHW plays a major role in enhancing anlotinib to inhibit tumor growth in LLC-bearing mice, and its efficacy was followed by that of the other three herbs. In fact, Shexiang is the king herb in XHW according to the compatibility principle “monarch, minister, assistant, and guide” of the prescription, which means Shexiang plays a primary role in achieving the best overall effect. Our experimental results verified that XHW and anlotinib synergistically decrease protein levels of PDGFRA.

Additionally, 2 components in Niuhuang, 8 components in Shexiang, 30 components in Ruxiang, and 60 components in Moyao acted on PLA2G1B, PLA2G2E, CES1, PPARG, PTGS1, ALOX5, ADRB1, ADRA1A, MAOB, ADRA2B, ADRB2, TAAR1, UGCG, and PTGS2 to modulate six metabolites directly. Phospholipase A (PLA) is present in many organisms and has semerged as a therapeutic target for cancer and some inflammatory diseases (31). High expression of the PLA2G1B gene is related to increased susceptibility to lung cancer in mice and humans (32, 33). PLA2G1B and PLA2G2E contribute to the release of hexadecanoic acid, linoleic acid, arachidonic acid and lysoPC [18:1(9Z)] and participate in the regulation of glycerophospholipid metabolism and amino acid metabolism. ADRA1A, ADRA2B, ADRB1, and ADRB2 are the major subtypes of the superfamily of G protein-coupled receptors named adrenergic receptors (ARs) (34). Many studies have shown that ARs are closely related to tumor cell invasion and migration (35). β-ARs are constitutively expressed in most mammalian cells. ADRB2 induces phosphorylation of ERK1/2 and CREB, thereby promoting the proliferation of A549 lung cancer cells (36). Furthermore, PTGS1,2 (37), PPARG (38, 39), MAOA (40), MAOB (41), ALOX5 (42–44), CES1 (45), TAAR1 (46), and UGCG (47) are involved in various metabolic processes of lung cancer and tumor resistance.

The network pharmacology results show that XHW improves the efficacy of anlotinib in LLC-bearing mice through multiple components and targets. In summary, the ingredients in XHW can act directly on the targets of anlotinib and regulate endogenous metabolic disturbances *in vivo*, thereby synergistically inhibiting tumor growth. Our results highlight the advantages of TCM combined with anlotinib in the treatment of lung cancer. However, it should be noted that further work is required to verify the relationship between these targets and metabolites and to clarify the underlying molecular mechanisms. This research provides new insights into the regulatory properties of XHW that improves the effect of anlotinib in the treatment of lung cancer through an integrated approach.

## Data Availability Statement

The original contributions presented in the study are included in the article/**Supplementary Material**. Further inquiries can be directed to the corresponding authors.

## Ethics Statement

The whole experiment was performed in accordance with the NIH Guide for the Care and Use of Laboratory Animals approved by the Scientific Investigation Board of Cancer Hospital, Chinese Academy of Medical Sciences and Peking Union Medical College.

## Author Contributions

CL, ZW, and CW performed the experiments, analyzed the data, and wrote the manuscript. BC and MZ collected and prepared samples. GQ, SQ, and XF performed the analyses. YS and XL amended the paper. RL, JW, and GL designed the study and amended the paper. All authors contributed to the article and approved the submitted version.

## Funding

This work has been financially supported by Beijing Municipal Science & Technology Commission (Z181100001618003) and CAMS Innovation Fund for Medical Sciences (CIFMS) (Grant no. 2016-I2M-1-001).

## Conflict of Interest

The authors declare that the research was conducted in the absence of any commercial or financial relationships that could be construed as a potential conflict of interest.

## Publisher’s Note

All claims expressed in this article are solely those of the authors and do not necessarily represent those of their affiliated organizations, or those of the publisher, the editors and the reviewers. Any product that may be evaluated in this article, or claim that may be made by its manufacturer, is not guaranteed or endorsed by the publisher.

## Supplementary Material

Supplementary Figure 1 PCA score plots of the combination of anlotinib with XHW against lung cancer in ESI- mode.

Supplementary Figure 2 Analysis of potential biomarkers associated with anlotinib plus XHW in the treatment of lung cancer in ESI- mode. **(A)** Results of the OPLS-DA model using the data from the anlotinib/XHW group *vs* the anlotinib group. **(B)** S-score plot constructed from the supervised OPLS analysis of the anlotinib/XHW group *vs* the anlotinib group. **(C)** Results of the OPLS-DA model using the data from the anlotinib/XHW group *vs* the XHW group in ESI+ mode. **(D)** S-score plot constructed from the supervised OPLS analysis of the anlotinib/XHW group *vs* the XHW group. Metabolite ions with VIP values >1 and |p(corr)| ≥ 0.5 are marked with a red square. **(E) **Venn diagram of metabolites showing the commonality and peculiarity between the anlotinib/XHW, anlotinib, and XHW groups.
