# Supplementary figures and images for: An Integrative Metabolomic and Network Pharmacology Study Revealing the Regulating Properties of Xihuang Pill That Improves Anlotinib Effects in Lung Cancer
Source: Front Oncol. 2021 Aug 9;11:697247. doi: 10.3389/fonc.2021.697247 (PMC8381607; doi:10.3389/fonc.2021.697247)

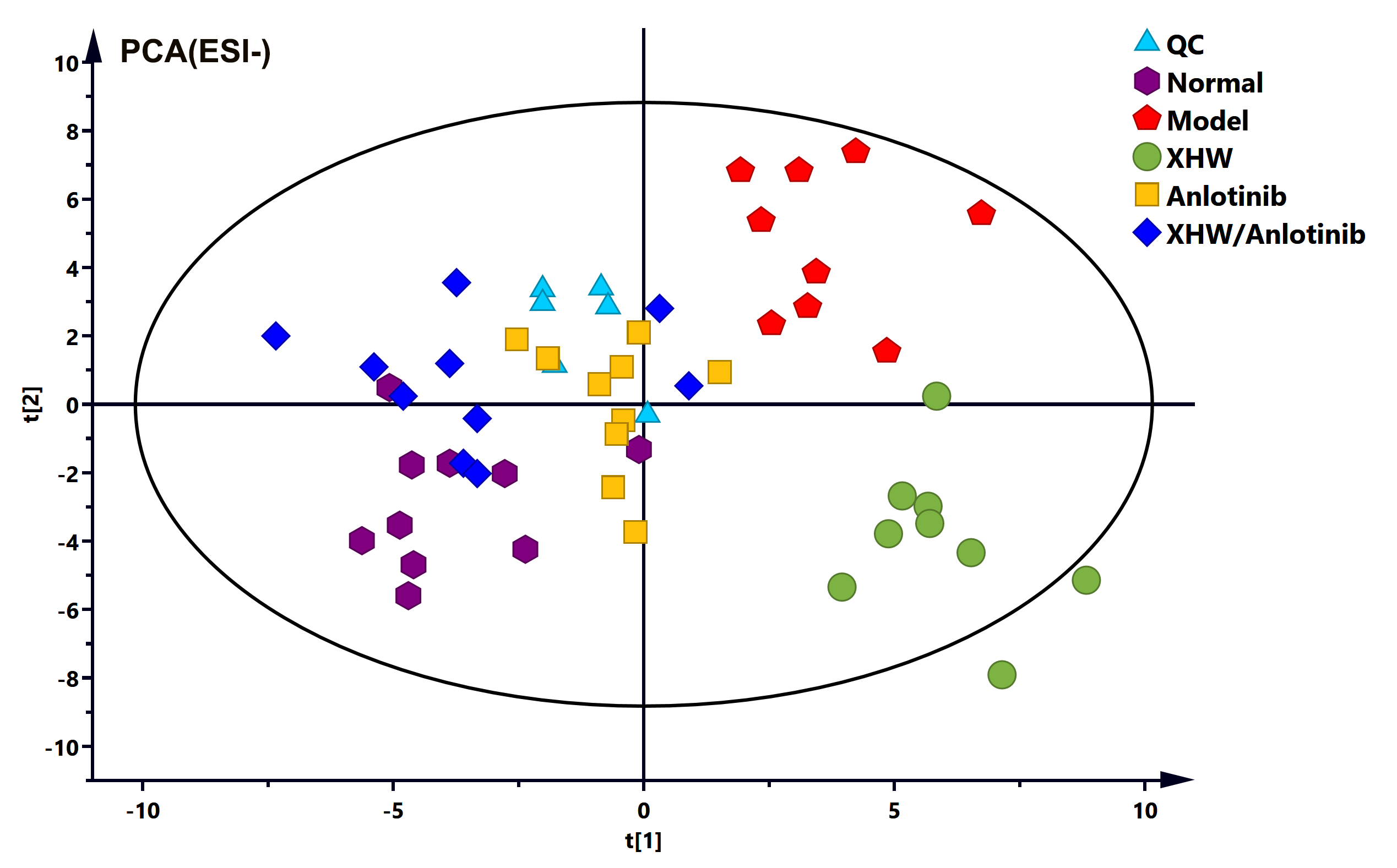

Supplement: Supplementary Figure 1 — PCA score plots of the combination of anlotinib with XHW against lung cancer in ESI- mode. [file Image_1.tif]

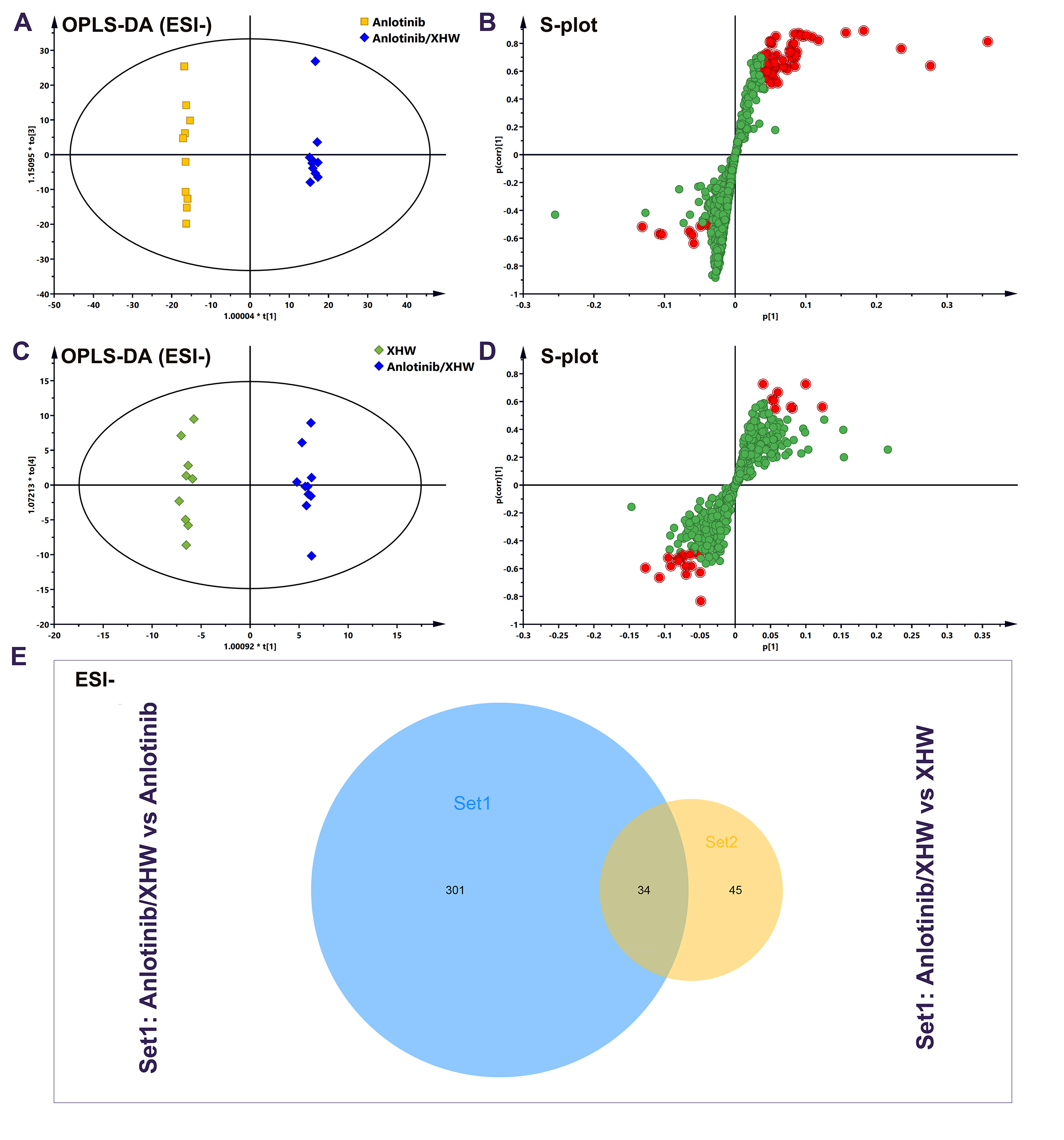

Supplement: Supplementary Figure 2 — Analysis of potential biomarkers associated with anlotinib plus XHW in the treatment of lung cancer in ESI- mode. (A) Results of the OPLS-DA model using the data from the anlotinib/XHW group vs the anlotinib group. (B) S-score plot constructed from the supervised OPLS analysis of the anlotinib/XHW group vs the anlotinib group. (C) Results of the OPLS-DA model using the data from the anlotinib/XHW group vs the XHW group in ESI+ mode. (D) S-score plot constructed from the supervised OPLS analysis of the anlotinib/XHW group vs the XHW group. Metabolite ions with VIP values >1 and |p(corr)| ≥ 0.5 are marked with a red square. (E) Venn diagram of metabolites showing the commonality and peculiarity between the anlotinib/XHW, anlotinib, and XHW groups. [file Image_2.tif]
