# Supplementary material for: An Integrative Metabolomic and Network Pharmacology Study Revealing the Regulating Properties of Xihuang Pill That Improves Anlotinib Effects in Lung Cancer
Source: Front Oncol. 2021 Aug 9;11:697247. doi: 10.3389/fonc.2021.697247 (PMC8381607; doi:10.3389/fonc.2021.697247)
Supplement: Supplementary file 4 [file DataSheet_2.zip › Supplementary Table 1.DOCX]

Supplementary Table S1 Illustration of a 4-point scoring system(1).

| Numerical score | Description | Definition |
| --- | --- | --- |
| 0 | Within normal limits | Tissue considered to be normal, under the conditions of the study and considering the age, sex, and strain of the animal concerned. Alterations may be present, which, under other circumstances, would be considered deviations from normal. |
| 1 | Minimal | The amount of change present barely exceeds that which is considered to be within normal limits. |
| 2 | Slight | In general, the lesion is easily identified but of limited severity. |
| 3 | Moderate | The lesion is prominent, but there is significant potential for increased severity. |
| 4 | Severe | The degree of change is as complete as possible (occupies the majority of the organ). |

References

1. Mann PC, Vahle J, Keenan CM, Baker JF, Bradley AE, Goodman DG, et al. International harmonization of toxicologic pathology nomenclature: an overview and review of basic principles. *Toxicol Pathol* (2012) 40(4 Suppl):7s-13s. Epub 2012/06/01. doi: 10.1177/0192623312438738. PubMed PMID: 22637736.
