# Supplementary material for: An Integrative Metabolomic and Network Pharmacology Study Revealing the Regulating Properties of Xihuang Pill That Improves Anlotinib Effects in Lung Cancer
Source: Front Oncol. 2021 Aug 9;11:697247. doi: 10.3389/fonc.2021.697247 (PMC8381607; doi:10.3389/fonc.2021.697247)
Supplement: Supplementary file 4 [file DataSheet_2.zip › Supplementary Table 2.DOCX]

Supplementary Table S2 Scores of histopathological sections of the heart, liver, spleen, lung and kidney of the mice bearing LLC after treatment for 21 days.

| Pathologists | Organs | Model | Anlotinib | XHW | Anlotinib/XHW |
| --- | --- | --- | --- | --- | --- |
| 1 | Heart | 0 | 0 | 0 | 0 |
| 2 | Heart | 0 | 0 | 0 | 0 |
| 3 | Heart | 0 | 0 | 0 | 0 |
| 1 | Liver | 0 | 0 | 0 | 0 |
| 2 | Liver | 0 | 0 | 0 | 0 |
| 3 | Liver | 0 | 0 | 0 | 0 |
| 1 | Spleen | 0 | 0 | 0 | 0 |
| 2 | Spleen | 0 | 0 | 0 | 0 |
| 3 | Spleen | 0 | 0 | 0 | 0 |
| 1 | Lung | 1 | 0 | 0 | 0 |
| 2 | Lung | 1 | 0 | 0 | 0 |
| 3 | Lung | 1 | 1 | 1 | 1 |
| 1 | Kindney | 0 | 0 | 0 | 0 |
| 2 | Kindney | 0 | 0 | 0 | 0 |
| 3 | Kindney | 0 | 0 | 0 | 0 |
